# Probability distributed time delays: integrating spatial effects into temporal models

**DOI:** 10.1186/1752-0509-4-19

**Published:** 2010-03-04

**Authors:** Tatiana T Marquez-Lago, André Leier, Kevin Burrage

**Affiliations:** 1 Department of Biosystems Science and Engineering, Swiss Federal Institute of Technology (ETH) Zurich, Mattenstrasse 26, CH-4058 Basel, Switzerland; 2 Swiss Institute of Bioinformatics, ETH Zurich, CH-8092 Zurich, Switzerland; 3 COMLAB, University of Oxford, OX1 3QD, UK; 4 Institute for Molecular Biology, The University of Queensland, Brisbane, QLD 4072, Australia

## Abstract

**Background:**

In order to provide insights into the complex biochemical processes inside a cell, modelling approaches must find a balance between achieving an adequate representation of the physical phenomena and keeping the associated computational cost within reasonable limits. This issue is particularly stressed when spatial inhomogeneities have a significant effect on system's behaviour. In such cases, a spatially-resolved stochastic method can better portray the biological reality, but the corresponding computer simulations can in turn be prohibitively expensive.

**Results:**

We present a method that incorporates spatial information by means of tailored, probability distributed time-delays. These distributions can be directly obtained by single *in silico *or a suitable set of *in vitro *experiments and are subsequently fed into a delay stochastic simulation algorithm (DSSA), achieving a good compromise between computational costs and a much more accurate representation of spatial processes such as molecular diffusion and translocation between cell compartments. Additionally, we present a novel alternative approach based on delay differential equations (DDE) that can be used in scenarios of high molecular concentrations and low noise propagation.

**Conclusions:**

Our proposed methodologies accurately capture and incorporate certain spatial processes into temporal stochastic and deterministic simulations, increasing their accuracy at low computational costs. This is of particular importance given that time spans of cellular processes are generally larger (possibly by several orders of magnitude) than those achievable by current spatially-resolved stochastic simulators. Hence, our methodology allows users to explore cellular scenarios under the effects of diffusion and stochasticity in time spans that were, until now, simply unfeasible. Our methodologies are supported by theoretical considerations on the different modelling regimes, i.e. spatial vs. delay-temporal, as indicated by the corresponding Master Equations and presented elsewhere.

## Background

Biological systems are characterized by complex spatial structure, low diffusion rates, or entail acute spatial dependencies, requiring spatially resolved simulations. Consequently, a system's behavior can vary considerably compared to its well-mixed representation, a fact that has been previously shown through spatially-resolved models [1-4]. In recent years, it has become evident that one must incorporate spatial aspects in a model in order to achieve two main purposes. First, to understand 'how and when' spatial processes play key roles within actual cellular processes, affecting their modeling outcomes and interpretation. Secondly, to learn how to incorporate such spatial effects in a reliable and accurate manner.

The most straightforward spatial technique is the representation of chemical kinetics through reaction-diffusion partial differential equations. However, this deterministic approach is only valid when dealing with large molecular concentrations and when noise is not amplified throughout the system. If at least one of these conditions fails to hold, one must rely on spatial stochastic simulators, which can be discrete or continuous and have different levels of spatial resolution [4].

Stochastic spatially-resolved simulations are, in general, very costly as compared with their solely temporal counterparts. By consequence, one should always keep in mind the trade-off between simulation time and level of resolution. The highly resolved end of the spectrum is represented by lattice and off-lattice particle methods [5-7]. Particle methods can provide very detailed simulations of highly complex systems at the cost of exceedingly large amounts of computational time and, possibly, restrictions on the size of the simulation domain. Hence, such detailed simulations can often only yield short simulation time spans that may not be of interest to the experimentalists.

An alternative to particle methods, albeit still computationally expensive in many scenarios, is the discretization of the Reaction-Diffusion Master Equation (RDME) into reactive neighboring sub-volumes. In [8] the authors provide the specific outline for extending discrete stochastic simulators to the RDME regime, while the algorithms in [9,10] provide clever extensions using the ideas behind the 'next reaction method' [11]. Furthermore, there is an algorithm that accurately coarse-grains the RDME [4], yielding considerably shorter computational times.

However, there are certain scenarios in which all of the above methods can still be computationally expensive, especially for long simulation time spans. It is at this point when one should remember that, by incorporating delays into temporal models, one can in principle account for myriads of microscopic steps [12,13], if the delays are posed correctly. In other words, by incorporating delays into a temporal model one can capture essential information on a macroscopic level, each delay encompassing sets of biochemical processes or transport and events on a microscopic time scale that would otherwise render us unable to compute cell dynamics in real-time. Some examples for the use of delays in modeling biochemical reaction networks can be found, for instance, in [14-16], where it can be readily observed that the consideration of delays is not only practical, but many times essential for capturing the dynamics accurately.

Having this in mind, we introduce a methodology that indirectly incorporates spatial features and effects into temporal models, by means of using tailored distributed delays in a discrete stochastic setting. This idea is compatible with modular or 'plug and play' models, a common concept used in synthetic biology [17-19], that we now propose to extrapolate to include spatial effects in arbitrary cellular processes. In our methodology each 'plug' would correspond to a delay distribution describing a diffusion-driven event that can be obtained from single suitable *in silico *or *in vitro *experiments. For the former, the single spatial simulations are relatively inexpensive as they only describe diffusion inside or between compartments for single events and not a whole process. Once the delay distributions for single events are fed into a delayed stochastic solver, they provide the 'raw material' necessary to obtain myriads of different stochastic trajectories, accounting for molecular motion at one or several stages. Ideally, these can describe a full downstream pathway or cellular process, where these diffusion profiles can allow us to explore variations in our model (such as the order or number of cellular events) or even study related/similar cellular processes and signaling pathways. Nevertheless, both translocational feedbacks (switching back and forth between compartments) and strongly-coupled delayed scenarios (i.e. several delayed reactions compete for the same reactants) may pose limitations in terms of accuracy, if using fixed distributions in the whole simulation timecourse, due to non-negligible effects from time-varying delay distributions. These topics will be described in detail in our Results section.

We apply our method to a variety of scenarios of molecular translocation and association processes, reaching a good compromise between accuracy and computational costs. Our simulations, as compared to those yielded by ChemCell (a single-particle tracking algorithm developed in Sandia National Laboratories [1], see Methods), show high accuracy while being computed several orders of magnitude faster. Additionally, we present a methodology based on delay differential equations that can be used in scenarios of high molecular concentrations and low noise propagation.

## Results

### New methodology for discrete stochastic simulations: dDSSA

Our methodology is composed of two steps: distribution fitting and stochastic simulation. The first step is crucial and will determine how accurate the method is compared to a highly resolved particle tracking method. The second step is achieved by using a generalization of the SSA for chemical kinetics with delays (DSSA) [12,20,21], where a constant delay is no longer considered, but a distribution from which individual delays are to be drawn. Initially, the reaction rate constant of a delayed reaction is set to a high value such that its waiting time is relatively small compared to the sampled delays. This condition is not necessary, and will be later on removed or replaced. Nevertheless, in simple translocation scenarios it can be used without loss of accuracy, achieving higher computational savings. For easier referencing, we will refer to this new methodology as dDSSA (distributed delay stochastic simulation algorithm).

### First step: Distribution fitting

Intuitively, if one has to assign a 'delay' for a certain process to happen, the first idea that may come to mind is to measure the duration of such event in each repetition of an experiment performed under 'identical' conditions. With this in mind, one may think of a 'diffusion delay' as the first passage time of a molecule into a predefined subset of the domain or possible chemical state. For instance, if a molecular species is initially localized in the cytosol but bound to translocate to the nucleus, one can measure how long each molecule takes to translocate, associate a delay to each arrival time, and draw statistics on it. Some experimental techniques that can be used for deriving such delay distributions are: real-time production of single protein molecules [22], GFP time-courses describing compartmental localization [23], or 'tagging' proteins with explicit localization signals (the most common of which are nuclear import and export). A second example might be measuring delays associated with dimer formation by measuring particle collision times. Even though such resolved data is often unavailable and one merely has an average parameter such as the mean-square displacement, the diffusion constant or the binding rate constant, one can still benefit from first passage abstractions [24,25] or stochastic simulations portraying random diffusion and directed transport.

For the purposes of this paper we obtained the delay distributions directly from single ChemCell simulations. To represent a diffusion dependent event (such as a translocation to a different compartment, or collision between two molecules) by means of a temporal delay, we generated appropriate probability and cumulative distribution functions (PDF and CDF, respectively). Namely, for a particular initial condition characterized by molecular concentrations and particle locations, one can record the time at which the next molecule performs the event in question, be it translocation or molecular collision, from which a certain CDF can be derived given that the sample of experiments is large enough. It is worth highlighting that the derivation of any delay distribution requires only a few (most times only one) relatively inexpensive spatial simulations, the results of which are fed into the DSSA algorithm yielding myriads of stochastic scenarios at 'solely temporal simulation' costs.

For translocation processes, any particle, at any time point is either already absorbed, i.e. inside its destination compartment, or its location can be described by *p*(, *t*). Hence, we can calculate the probability distribution for arrival (absorption) before or at time *t *as

where we integrate over the volume *V *in which the particle is diffusing. For the delay stochastic simulation algorithm we only need this CDF, *P*(*t*_*a *_≤ *t*), of the delay/arrival time distribution. However, it should be noted that the PDF of arrival times is given by *dP*(*t*_*a *_≤ *t*)/*dt*.

Alternatively, in scenarios of high molecular concentrations, delay distributions of simple translocation processes can also be obtained by solving the more general advection-diffusion equation,

subject to appropriate boundary and initial conditions and characterized by the particles' diffusion constant *D *and the geometry of the spatial domain. Here, the advective term is only needed when modelling diffusion processes with directed transport. The boundary conditions are usually mixed Neumann-Dirichlet conditions corresponding to reflective and absorbing boundaries, depending on the specific geometry of the problem. For instance, diffusion from inside the nucleus to the cytoplasm, diffusion from the cytoplasm into the nucleus, or diffusion from the extracellular matrix into a cell all have distinctive boundary conditions that allow for 'driving' molecular directionality. In the case of translocation between different compartments, there is always at least one boundary condition that describes the absorbing barrier, for example that of the membrane separating the 'donor' from the 'receiving' compartments.

Analytic solutions to the diffusion equation can be obtained, albeit rarely and many times under a variety of simplifying conditions regarding the domain geometry, the initial and boundary conditions. Actually, for many relevant applications, analytic closed solutions are simply impossible to obtain. In these cases we can use numerical techniques that approximate the real solution with a maximal error up to a predefined user-specified value. For instance, one could numerically solve the diffusion equation on an arbitrary domain using pre-compiled software, such as COMSOL (or equivalent), or opt for a tailored discretization technique, using finite differences or finite elements.

**Figure 1:**
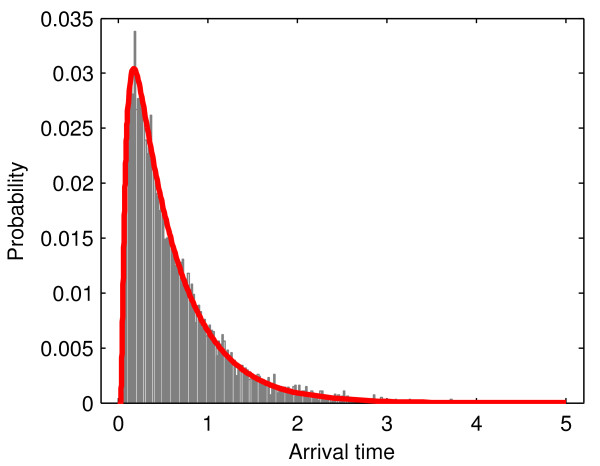
**Distribution of particle absorption times in a 1D discretized space**. The interval has size 3.52 μm. Initially a particle starts in 0.81 μm distance to the right boundary and diffuses with step-size 0.001 μm. The left boundary (at 0) is absorbing while the right boundary is reflecting. The normalized histogram over 10,000 random walks showing the number of particles that are absorbed within time intervals of 0.001 seconds is showed in gray. The corresponding numerical solution of *dP*(*t*_*a *_≤ *t*)/*dt *is plotted as a solid red line.

In many cases, molecular concentrations within the cell are very low [12,13], in which case a deterministic representation of diffusion will not suffice. It is here where one must rely on sample particle trajectories (from either *in silico *or *in vitro *experiments) in order to construct a probability distribution that characterises particle's movement in a more reliable way. As an illustrative example, Figure 1 shows a normalized histogram of arrival times to a predefined absorbing boundary, obtained from 10,000 random walks in a 1D discretized interval. The corresponding numerical solution of *dP*(*t*_*a *_≤ *t*)/*dt *is plotted as a solid line showing that, as the molecular populations grow larger (or, equivalently, the number of random walks becomes large), the normalized histogram will converge to the PDF of arrival/absorption times.

### Second step: model building and stochastic simulation with delayed reactions

In terms of our temporal framework, compartments are introduced via additional species such that identical biological species are distinguished according to the compartment where they are localized. Translocation processes are then modelled as delayed unary reactions whereas bimolecular reactions, i.e. associations of two molecules, depend on how fast these molecules diffuse and their reaction radius. Another novel feature of our methodology is that, since bimolecular reactions are diffusion-driven, they are also modelled by incorporating a delay. This is particularly useful when accounting for low diffusion rates, anisotropies, or spatial patterns far away from well-mixedness.

Several delay stochastic simulation algorithms have been developed in order to take account of intrinsic noise and delays associated with reactions [12,20,21,26]. Here, we extend the DSSA by Barrio et al. (see Methods and [12]) but it should be noted that other DSSA implementations might be equally suitable, assuming they account for 'consuming reactions' (such as [20,21]).

In order for the DSSA to be applicable, we adapted the algorithm such that delays are no longer considered to be constant but are actually drawn from the CDFs derived in the first step (*modification ****M1***). The associated reaction rates are all set to an arbitrary, high value, ensuring that the waiting times are rather small compared to the delays. However, one should pay careful attention while doing so, as this might add a bias towards delayed reactions in systems with competing reactions. For such, a further modification will be introduced later in this paper.

For the purpose of incorporating spatial effects, all delayed reactions are considered as consuming. In the original DSSA implementation [12] this implied that once a delayed reaction was drawn the corresponding reactants were no longer available for any future reactions, in order to not violate conservation of mass. There are two considerations at hand. First, that delayed translocation reactions may compete with other non-delayed reactions for the same reactants. Second, the reaction rates for delayed reactions are much larger than those for non-delayed reactions. All together, when choosing a delayed reaction, removing the reactants from the system would highly bias the dynamics towards diffusion.

In order to account for this, the DSSA was further modified, such that reactants that are assigned to diffusion/translocation reactions can still be chosen as reactants in other non-delayed reactions. In this case the translocation reaction will be cancelled and replaced by the non-delayed reaction (*modification ****M2***). In this way, competition between a delayed translocation reaction and a non-delayed reaction can still be accurately modelled. It should be noted that this is not equivalent to treating translocation delays as non-consuming reactions.

As will be shown below, this approach loses its compensating effect in the case of two (or more) delayed reactions that are competing for a common reactant. This is an effect of setting all rate constants for delayed reactions to an arbitrary high value, such that the waiting time to the next reaction becomes rather small compared to the delay. As a consequence, two competing delayed unary reactions will have identical propensities. However, in the case of competition with/between binary reactions, the difference in the reactions' propensities is exclusively due to the number/s of molecules from reactant species other than the common reactant. Such larger numbers of molecules imply that the corresponding delayed reaction is preferentially occurring, regardless of diffusion rates, spatial inhomogeneities, and other effects. This is rather unrealistic and in such scenarios the standard DSSA approach will fail in capturing the reaction dynamics properly. Allowing reactants to switch the delayed reaction they are participating in (*modification ****M2***), would slow down the simulation due to 'indecisive' reactants (i.e. reactants that switch multiple times before being eventually consumed) and would still not capture correctly the biophysical nature of the diffusion-driven association processes.

In order to tackle these limitations we propose an additional, yet more radical, modification to the original DSSA (*modification ****M3***). This modification is only applicable to scenarios solely composed by sets of delayed reactions, and comes along with a conceptual change in the way the simulation advances in time. Here, the selection of reactions will be based on their delay distributions instead of their propensities. Namely, for each possible reaction a delay is drawn from its corresponding delay distribution and, for each subset of coupled reactions, the minimum of the corresponding delays determines the reaction that is chosen to occur in the future. Bear in mind that all delayed reactions are consuming and, hence, once a reaction is chosen, its reactants are taken out of the pool of available molecules. Once no more reactions are possible, the simulation continues at the time of the next delayed reaction update.

Generally, deviation of the temporal approximation from the spatiotemporal dynamics can also be due to the time-variant spatial configuration of molecules in the cellular compartments and would require state-/time-dependent delay distributions. This is not a shortcoming of the simulation algorithm, as drawing from a state-dependent distribution does not involve substantial changes in the implementation, but rather an impracticality of the methodology, given the efforts needed to obtain multiple state-dependent delay distributions. However, depending on the reaction network to be modelled, the approximation can already be improved considerably by using piecewise delay distributions with only very few steps. In other words, appropriate delay distributions will be considered during selected time windows throughout the simulation. Each of these distributions is now able to capture the underlying delay mechanisms more accurately, be it for spatial inhomogeneities or abrupt changes in molecular concentrations. We refer to any form of time or state-dependent selection of delay distributions as *modification ****M4***.

### Test cases

**Figure 2:**
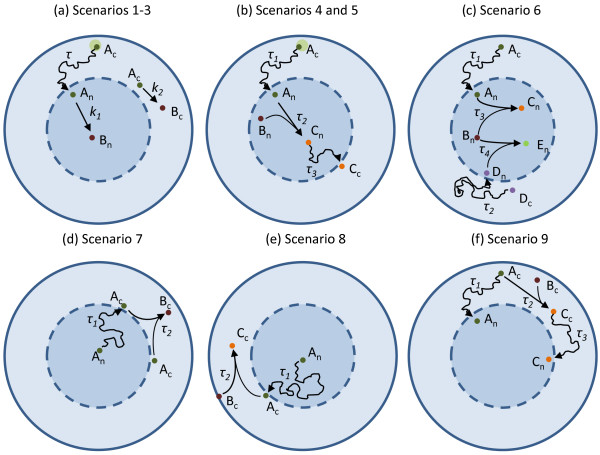
**Principal simulation scenarios considered in this study**. (a) Scenarios (Sc.) 1-3: nuclear translocation of particles *A*_*c *_(Sc. 1), followed by a unary reaction *A*_*n *_→ *B*_*n *_(Sc. 2) and the translocation reaction competing with the unary reaction *A*_*c *_→ *B*_*c *_(Sc. 3). (b) Sc. 4-5: nuclear translocation of *A*_*c *_followed by a nuclear binary reaction *A*_*n *_+ *B*_*n *_→ *C*_*n *_(Sc. 4) followed by the cytoplasmic translocation of the product *C*_*n *_(Sc. 5). (c) Sc. 6: upon translocation molecules *A*_*n *_and *D*_*n *_compete for the same binding partner *B*_*n *_(*A*_*n *_+ *B*_*n *_→ *C*_*n *_and *D*_*n *_+ *B*_*n *_→ *E*_*n*_) (d-e) Sc. 7-8: upon translocation molecules *A*_*c *_are able to dimerize (Sc. 7) or bind to a species initially localized in the cell membrane (Sc. 8). (f) Sc. 9: upon translocation molecules *A*_*c *_dimerize with molecules *B*_*c *_and their product *C*_*c *_is able to translocate back to the nucleus. In all corresponding temporal models of Sc.1-9, each delay distribution accounts for the spatial effects due to the diffusion of particles.

Here, we will explore ten different reaction-diffusion scenarios (see Figure 2 for Scenarios 1-9) to show both the applicability and limits of our methodology. The spherical geometry adopted in all scenarios assumes a cell and nucleus radii of 7.81 μm and 4.29 μm, respectively. These are typical values for a human carcinoma cell [27], but it should be noticed that our methodology is not restricted to this geometry since the delay distributions capture arrival times stemming from (and consequently portraying) arbitrary geometries. We further try several translocation and reaction profiles, as defined by different diffusion rate constants and initial spatial conditions (localized versus well-mixed). Initial spatial conditions were explored by comparing well-mixed particles inside the cytosol to particles localized in a cluster 'far away' from the nucleus, with a radius of 0.5 μm and localized 7 μm from the cell centre. All ChemCell simulations were performed with a uniform time step of 10^-3 ^seconds (or 10^-4 ^secs. in high diffusibility cases), a bin size described by the fastest diffusion rate (10^-7 ^cm^2^/sec), a reaction radius described by a maximal probability of 0.5 and cube Brownian motion for all particles. The latter imposes all particles' new positions to be sampled from Gaussian distributions truncated to fit within a cube surrounding their current position, the size of which is determined by the diffusion coefficient of the molecule (see Methods and [1]).

We also explored different membrane permeability scenarios, that is, probabilities with which a particle will enter a different compartment once localized in close proximity to its boundary. For all ChemCell simulations shown here we set permeability of the nuclear membrane to 100%. However, we benchmarked our methodology by studying changes of the permeability, directly reflected in the delay distributions, and our simulations yielded equally accurate results (data not shown).

In order to illustrate the overall accuracy of the temporal approximation we calculated a total relative error at each time point. Namely, the sum of the absolute differences between two simulations (ChemCell and dDSSA) over all species, divided over the total number of molecules in the system. It should be noted, however, that in some applications one might be concerned with the error in a particular species, as opposed to the reported total relative error (which can be considered an upper bound with respect to the former). For other specificities in each considered scenario, we refer to the captions of the corresponding simulation plots. All dDSSA simulations were performed until a steady state was reached and include either *modification ****M1 ***(with or without *modification ****M2***) or *modification ****M3 ***(with or without *modification ****M4***).

**Figure 3:**
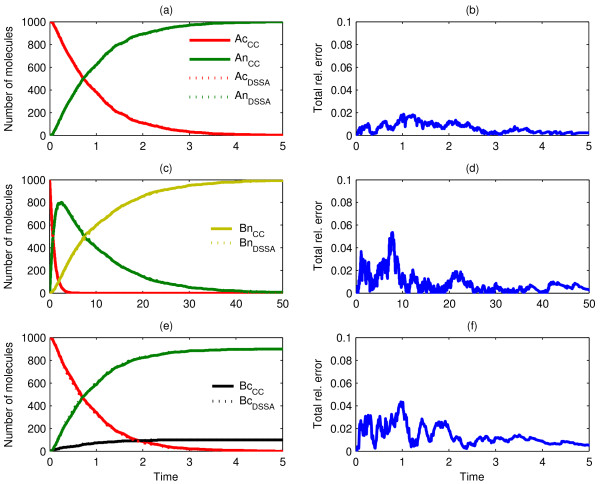
**Comparison of dDSSA and Chemcell: Scenario 1**. Single run (a, c) and mean of ten runs (e) of ChemCell as compared to the mean behaviour of ten independent dDSSA trajectories (a, c, e) with corresponding total relative error (b, d, f) for Scenario 1 (a, b), with diffusion constants D_Ac _= 10^-7 ^cm^2^/sec, Scenario 2 (c, d), with D_Ac _= 10^-7 ^cm^2^/sec and a reaction rate constant of *k = 0.1 s*^-1^, and Scenario 3 (e, f), where D_Ac _= 10^-7 ^cm^2^/sec and the competing reaction *A*_c _→ *B*_c _has a reaction rate constant *k = 0.1 s*^-1^.

Our first three Scenarios are schematically shown in Figure 2a. These portray simple nuclear translocation mechanisms for 1000 clustered *A*_*n *_particles inside the cytosol (Scenario1), along with a subsequent unary reaction inside the nucleus (Scenario 2) and the possible competition between nuclear translocation and a unimolecular reaction in the cytosol (Scenario 3). Single ChemCell runs (one for each scenario) resulted in arrival statistics from which the corresponding CDFs were calculated, subsequently fed as delay distributions to the dDSSA algorithm. As shown in Figure 3(a-f), dDSSA trajectories perfectly match ChemCell dynamics, where it is worth noting that including *modification ****M2 ***enabled us to accurately capture the competition between a unary reaction and a delayed translocation reaction in the purely temporal model (Fig. 3e, f). Additionally, it should be noted that a subsequent reaction taking place inside the nucleus is not affected by possible error accumulation from drawing random translocation delays as opposed to tracking single molecules.

Scenarios 4 and 5 are schematically shown in Figure 2b. They portray the nuclear translocation of 1000 clustered *A*_*n *_particles inside the cytosol, followed by a subsequent binary reaction taking place inside the nucleus (Scenario 4), and a possible further translocation of the product back to the cytosol (Scenario 5). In these cases, we split the problem into several distinct delayed-steps and obtained the corresponding delay distributions τ_1_,...,τ_N _for each process, from separate ChemCell runs. This is a necessary step when including binary reactions, and which we will refer to as *'delay splitting'*. In this case, the nuclear localization statistics from the full scenario simulation led to τ_1_. In order to derive τ_2 _we 'froze' in space the location of each *A*_*n *_as they entered the nucleus. We then used these coordinates as initial condition for the particles *A*_*n*_, which were set to diffuse and associate with *B*_*n*_, while the timing of each of these events was recorded yielding the CDF for τ_2_. Lastly, we computed τ_3 _by recording the translocation times of *C*_*n*_, by running ChemCell with an initial spatial location obtained from the full scenario simulation, as each association of *A*_*n *_and *B*_*n *_yielded a product *C*_*n*_. These precautions were taken as the initial position of *A*_*c *_was clustered inside the cytosol, which largely biases a uniform entry to the nucleus, and all events therein.

**Figure 4:**
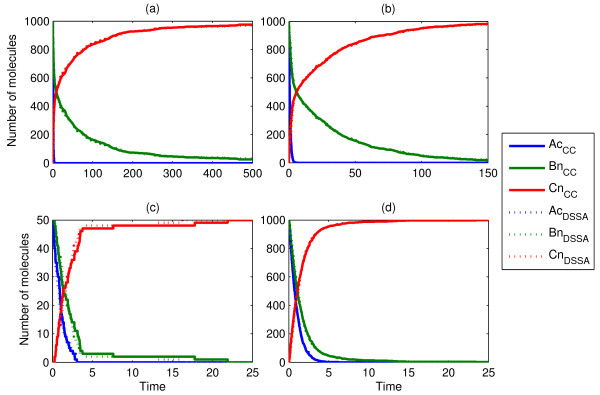
**Single runs of ChemCell and dDSSA trajectories, portraying Scenario 4**. Here, we considered several diffusion constants: (a, b) D_Ac _= D_An _= 10^-9 ^cm^2^/sec, (c, d) D_Ac _= D_An _= 10^-7 ^cm^2^/sec, (a, c, d) D_Bn _= 0 cm^2^/sec, (b) D_Bn _= 10^-9 ^cm^2^/sec. The last two examples (c, d) differ in the number of initial molecules *A*_*c*_, in order to show that lower molecular concentrations can also be accurately captured with our method.

**Figure 5:**
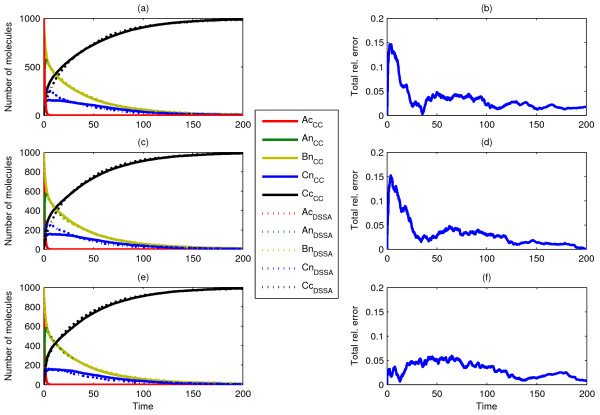
**Comparison of ten independent ChemCell and dDSSA simulations, portraying Scenario 5**. The three delay distributions are obtained from three separate ChemCell runs (delay splitting) with diffusion constants D_Ac _= 10^-7 ^cm^2^/sec and D_An _= D_Bn _= D_Cn _= 10^-9 ^cm^2^/sec. (a, b) dDSSA with modification ***M1***, (c-f) dDSSA with modification ***M3***, and (e, f) along with modification ***M4***, truncating the delay distribution for *C*_*n *_→ *C*_*c *_at 0.5 for the first time unit.

Evidently, the CDF for the delay in the association reaction, τ_2_, introduces some errors to the stochastic simulation as the statistics are obtained under the simplifying assumption that all *A*_*c *_enter the nucleus at the same time. Ideally, we would have a delay distribution for each possible configuration of *A *and *B *in the nucleus. However, obtaining this *in silico *would be very time consuming, while *in vitro *it would currently be unfeasible. Nonetheless, our simulations (Fig. 4 and 5a-d) reveal that, even though τ2 is only an approximation of a set of state-variant distributions, the resulting dDSSA trajectories with *modifications ****M2 ***and ***M3 ***match equally well those obtained from single particle tracking *in silico*. The approximation can be improved for the initial phase of the simulation by applying *modification ****M3 ***in conjunction with M4 (Fig. 5e, f). Here, we simply restrict the drawing of uniform random numbers for reaction *C*_*n *_→ *C*_*c *_within the first time unit to the interval [0, 0.5), effectively limiting the delays to ~[0, 4). Evidently, at the very beginning of the simulation, changes in the spatial configuration of the nucleus happen more rapidly than later on, as the first translocated molecules *A*_*n *_have a much higher chance of reacting with their binding partners *B*_*n*_. By truncating the delay distribution we restrict the drawing to small delays and, this way, emulate the initially faster dynamics. Needless to say, the use of additional delay distributions for certain spatial configurations encountered during simulation might lead to an even better approximation.

In Scenario 6 (Figure 2c) we explored the issue of competing delayed reactions stemming from two molecular species, *A*_*c *_and *D*_*c*_, entering the nucleus and binding to the same partner species *B*_*n*_. As was mentioned before, such a scenario may pose challenges for our initial methodology as an effect of setting all rate constants for delayed reactions to a certain high value. This comes in contrast to all previously considered test cases (as they do not include any competing binding reactions), where the delayed reactions rate constants could be fixed to an arbitrarily high value, or where using *modification ****M3 ***resulted in enhanced accuracy. The reason behind these shortcomings comes down to highly variable delay distributions, for which better approximations are yet to be derived.

**Figure 6:**
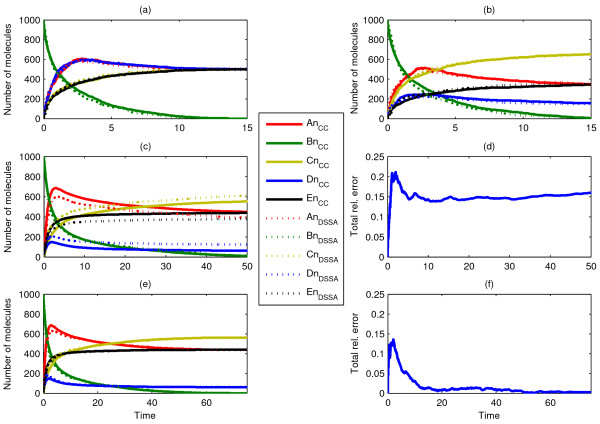
**Comparison of ChemCell and dDSSA trajectories, portraying Scenario 6**. Here, delay distributions are obtained from separate ChemCell runs (delay splitting) for each of the two translocation reactions and the competitive binding reactions. Diffusion rates are D_cyt _= 10^-9 ^cm^2^/sec and D_nuc _= 10^-9 ^cm^2^/sec for cytoplasmic and nuclear species, respectively. Cases correspond to different initial conditions: (a) 1000 *A*_*c *_and 1000 *D*_*c *_molecules uniformly distributed in the cytosol; (b-d) 1000 *A*_*c *_and 500 *D*_*c *_molecules (b) uniformly distributed in the cytosol or (c, e) *A*_*c *_is clustered and *D*_*c *_is well mixed, while (d, f) show their corresponding total relative error. All four cases start with 1000 Bn molecules uniformly distributed in the nucleus. In (e, f) we use a modified rate *k *= 0.015 for *A*_*n*_+ *B*_*n *_→ *C*_*n *_to increase accuracy. To facilitate reading, plots only show nuclear species.

Nevertheless, in order to analyze under which circumstances our methodology can faithfully reproduce the dynamics proposed in Scenario 6, we computed the delay distributions τ_1_, τ_2_, τ_3_, and τ_4 _from three separate ChemCell runs using 'delay splitting'. Our approach yielded good approximations of the ChemCell dynamics when both translocating particles *A*_*c *_and *D*_*c *_are set to be uniformly distributed in the cytosol (Figure 6a, b). However, in the scenario where *A*_*c *_is initially clustered and *D*_*c *_is uniformly distributed in the cytosol, the delay distributions for the two nuclear association reactions do not reflect the different spatial configurations of *A*_*n*_, *B*_*n*_, and *D*_*n *_that occur during a fully spatial simulation. In such scenario our approach based on *modification ****M1 ***(Figure 6c, d) does not produce accurate trajectories.

As was mentioned above, a better approximation could possibly be gained by state-variant delay distributions. Obtaining this information is, for obvious reasons, rather time consuming. However, one might be able to balance the effect of time-/state-varying delay distributions by tuning the reaction rate of the dominant delayed reaction, and this can even be done in a simple 'trial and error' manner. Figures 6e, f show that with such tuning (in this case choosing *k *= 0.015 s^-1^), one can obtain good simulation results: the steady states are almost perfectly matched (+/- 2 molecules), only in the first ten seconds can larger differences of about 100 molecules between ChemCell and dDSSA simulation be observed for *A*_*n*_, *B*_*n*_, and *C*_*n*_. However, it should be noted that such fitting of parameters is only possible on a case-by-case basis, and is independent of our methodology, as with any other kinetic rate optimization technique.

Scenarios 7 and 8 are schematically shown in Figure 2d and 2e. Here, nuclear *A*_*n *_has to translocate first to the cytosol (becoming *A*_*c*_) in order to be able to dimerize (Scenario 7) or bind to a species initially localized in the cell membrane (Scenario 8), respectively. For both scenarios we used 'delay splitting' and obtained two separate delay distributions from ChemCell, one for the translocation reaction, the other for the homodimer/heterodimer formation.

**Figure 7:**
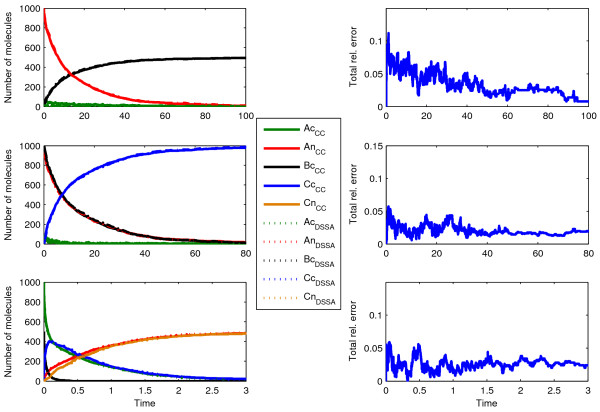
**Comparison of ChemCell and dDSSA simulations, portraying Scenarios 7 to 9**. (a, c, e) Single runs of ChemCell and dDSSA trajectories and (b, d, f) total relative error when compared with ChemCell. Cases correspond to Scenarios (a, b) 7 (c, d) 8 and (e, f) 9. ChemCell runs with diffusion rates of D_cyt _= 10^-7 ^cm^2^/sec and D_nuc _= 10^-9 ^cm^2^/sec for cytoplasmic and nuclear species, with the exception of D_Bc _= 10^-9 ^cm^2^/sec in Scenario 8. The considered initial conditions correspond to well-mixed molecules inside the corresponding compartment: (a) *A*_*n *_= 1000, (c) *A*_*n *_= *B*_*c *_= 1000, (e) *A*_*c *_= 1000 and *B*_*c *_= 500.

In Scenario 9 (Figure 2f) the translocation of *A*_*c *_to the nucleus competes with a binary reaction, the product of which is also able to translocate to the nucleus. In this case, we again used 'delay splitting' to obtain the delay distribution for translocation of the product to the nucleus. However, the delay distribution for the association reaction *A*_*c *_+ *B*_*c *_→ *C*_*c *_was easily inferred from the time course of *B*_*c*_. Simulation results for Scenarios 7-9 are shown in Figure 7(a, c, e).

**Figure 8:**
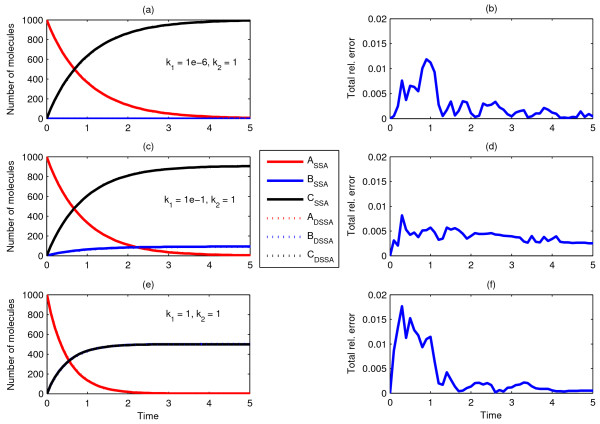
**Comparison of ChemCell and dDSSA simulations using modification *M3*, portraying Scenario 10**. Mean behaviour of 20 dDSSA (modification ***M3***) trajectories and relative error compared to the mean of 20 SSA runs for Scenario 10. The delay distributions were separately obtained from the mean trajectories of 20 SSA runs simulating *A *→ *X *with three different rates, *k *= 1, 1e-1, and 1e-6.

Lastly, in Scenario 10 (not shown in Figure 2) we wanted to explore the dynamics of two competing unary reactions, *A *→ *B *and *A *→ *C*, with reaction rates *k*_*1 *_and *k*_*2*_, respectively, in the form of delay distributions instead of driven by their respective rate constants. In contrast to Scenario 3, each unimolecular reaction, although not driven by diffusion, has a specific delay distribution assigned. Given the lack of dependency on diffusion, the delay distributions were obtained from SSA runs (assuming well mixedness), while simulating each reaction separately. We generated delay distributions for three different reaction rates *k*_*1 *_= 1e-5, 1e-1, and 1 and compared mean dDSSA with mean SSA behaviour for initial A(t = 0) = 1000 and *k*_*2 *_= 1. As could be expected, our methodology with *modification ****M1 ***was bound to fail due to the choice of reaction rates for delayed reactions and the way reactions are selected (data not shown). However, *modification ****M3 ***mimics perfectly the SSA dynamics for all three values of *k*_*1*_, as is shown in Fig. 8(a-f).

In summary, one can observe that, in the absence of acute changes in delay distributions, both ChemCell and our methodology yield strikingly similar results. However, in the case of our method (and any modification therein), the delay distributions were obtained from a handful of spatially resolved, albeit less computationally costly, runs, highlighting the fact that numerous stochastic trajectories portraying accurate average dynamics can also be obtained from single translocation profiles, at much lower computational cost.

### Comparison to deterministic models and a novel DDE method

Lastly, it is important to ask: how well is a purely temporal deterministic model able to mimic the spatiotemporal dynamics of such rather simple reaction networks? To answer and illustrate this question let us focus on Scenario 5, for which we shall follow the standard ODE approach and set up a system of five ODEs modelling translocation of *A*_*c *_and *C*_*n *_as unimolecular reactions with associated rate constants.

**Figure 9:**
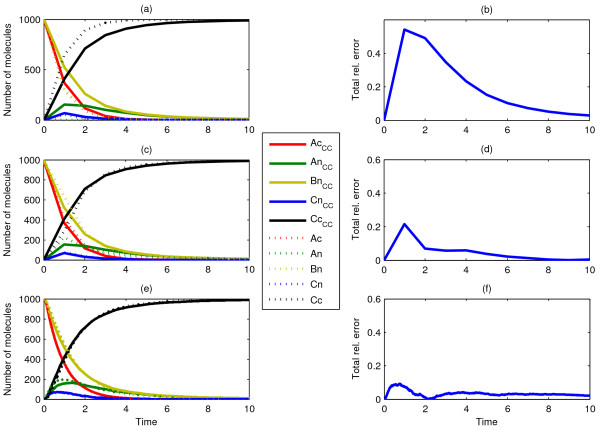
**Comparison of dDSSA and deterministic solutions of Scenario 5**. The reaction rates in deterministic solutions were estimated through an evolutionary strategy, to fit the average of ten ChemCell simulations. (a, c, e) ODE, DDE, and dDSSA (M3) model dynamics as compared with ChemCell; (b, d, f) show the corresponding error plots. Diffusion rates are D_Ac _= D_An _= D_Cn _= 10^-7 ^cm^2^/sec, D_Bn _= 0 cm^2^/sec in all cases.

Parameters of all three reactions were estimated with an evolutionary strategy where, as a fitness function, we used the least square error between the solution and the average of ten ChemCell runs for a sample set of time points. Figure 9a shows the system dynamics for the best evolved parameter set after 100 generations over 10 evolutionary runs. As was to be expected it becomes apparent that the ODE system is unable to match the dynamics obtained from the spatial simulator, the best example of which is species *A*_*n*_, which remains to be close to zero in the ODE solution. The total relative error is indicated in Figure 9b.

In order to resolve this issue for systems where molecular concentrations are relatively large, as a next step we propose a novel delay differential equation (DDE) methodology for solving problems with subsequent translocations and intermediate reactions, such as Scenario 5. In order to mimic consuming reactions, for each delayed reaction in the dDSSA model an associated buffer variable (B_1_-B_3_) in the DDE model was introduced. The full model is then described by the following equations:

Note that each molecular species in the dDSSA is now related to itself and the corresponding buffer in the DDE methodology. For instance, A_*c*_ in dDSSA is related to A_*c *_and B_1 _in the DDE model. However, ODEs stiffness, due to widely differing eigenvalues, is a general problem when trying to estimate parameters using evolutionary algorithms as the process can rapidly become very inefficient.

In our case, due to acute stiffness and the introduction of delay parameters as variables, we first estimated all nine parameters (rate constants k_1_- k_6 _and the delays τ_1_-τ_3_) manually, namely by changing single parameters one by one. Upon finding a reasonably good initial parameter set, we used the evolutionary strategy and fitness function described above for fine-tuning. Figures 9c and 9d display the system dynamics for the best evolved parameter set after 100 generations over 10 evolutionary runs and the corresponding relative error. As could be expected, the DDE model has lower errors than the ODE model and the dDSSA model performs best (Figures 9e and 9f), although not significantly better than the DDE model. It should be noted that this is the case given the high molecular concentrations, while for smaller numbers of molecules and/or greater noise sensitivity one should expect greater differences between the DDE and dDSSA methods. Nevertheless, the DDE approach can be useful in its own right when dealing with large numbers of molecules (when the DSSA becomes naturally slow) or as part of a hybrid algorithm.

### A brief discussion on computational costs

Evidently, the highly resolved spatiotemporal stochastic simulations with ChemCell are computationally more costly than the purely temporal stochastic simulations using the dDSSA. All dDSSA simulations are several orders of magnitude faster than ChemCell, depending on the number of reactions and molecules, cellular and nuclear volume, and diffusion constants of the molecular species. For instance, a single simulation of Scenario 5 representing 500 seconds of real-time dynamics takes more than 15 minutes on an Intel Core2 Quad processor system (Q6600, 2.4 GHz) when using ChemCell, while the dDSSA with *modification ****M1 ***takes only about 0.3 seconds on a computer with Intel Core 2 Duo CPU (T9300, 2.5 GHz). This would mean that for many reaction-diffusion scenarios one can roughly expect three orders of magnitude shorter computation times. Moreover, our dDSSA implementation is currently written in Matlab, while ChemCell is implemented in C. This is worth noting as one generally expects considerable speedups for codes implemented in C as compared to Matlab (usually by several orders of magnitude). In view of the huge difference in simulation times and coding language, we omit a detailed comparison of runtimes. However, one can foresee where the large gap between computation times stems from, and the speed-up one in principle could expect. Especially in the scenario of low numbers of reacting molecules diffusing in large volumes and/or with slow diffusion rates reactions will rarely occur. Hence, ChemCell (or any other particle simulator) will spend a large proportion of their runtime on diffusion steps without any reactions happening.

## Discussion

We have introduced two temporal-methodologies that incorporate spatial effects accurately, by means of probability distributed delays and/or particle buffers. Furthermore, we showed that our method's accuracy is exceptionally good for a wide range of scenarios incorporating chemical reactions and explicit molecular translocation between compartments. However, certain scenarios might pose additional challenges that require special treatment, such as kinetic rate transformation, introduction of artificial species or combination with other techniques (such as spatial SSAs), to further increase accuracy. These critical scenarios refer to cases in which the delay distributions are time dependent.

For instance, particles that are initially in a specific spatial configuration might diffuse and, when returning to their original compartment, create a significantly different spatial configuration. Such 'feedbacks' require a time-varying delay distribution profile. Two ways to account for this are analytical abstractions and/or the introduction of intermediate-step artificial species in the simulation, as described in *modification ****M4***.

**Figure 10:**
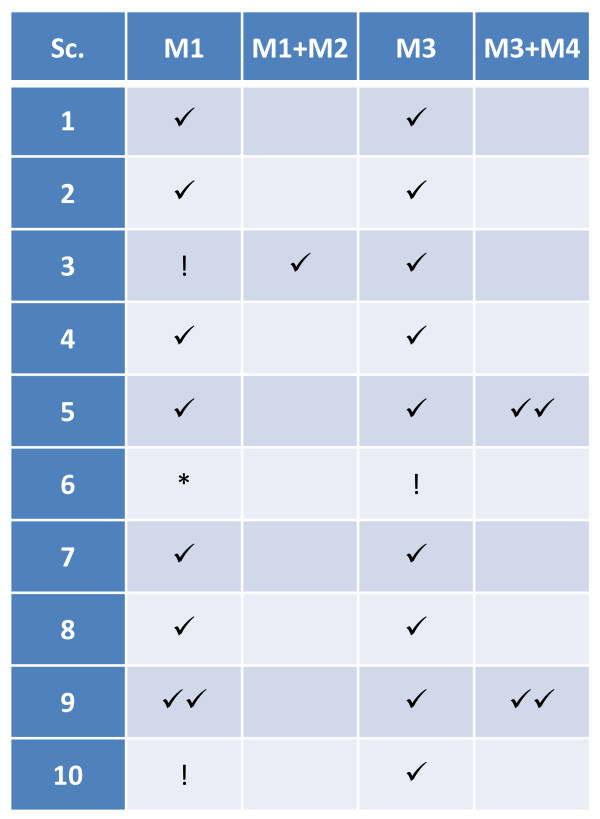
**Overview of test scenarios and corresponding algorithmic modifications**. Legends correspond to algorithmic modifications where dDSSA: (✔) matches trajectories obtained from ChemCell reasonably well, (✔✔) matches trajectories obtained from ChemCell best, (!) fails to accurately simulate the dynamics, or (*) matches trajectories obtained from ChemCell upon rate tuning.

We speculate that another feasible approach is to draw two random numbers, first the particle position (for instance, the distance to the nuclear centre) and, secondly, the associated delay from a position-dependent delay distribution. However, if molecules are not well-mixed within a confined compartment, obtaining adequate spatial distributions will entail additional costs. This topic requires further study, as well as the construction of a general parameter, possibly based on reaction coupling, that indicates which modification to use in which particular setting (current work in progress). In all scenarios presented here, the selection of a method/modification was straightforward, but this may not always be the case. A summary of all methodologies applicable to our simulation scenarios can be found in Figure 10.

Despite the limitation observed in cases where the delay distributions are time dependent, our methodology provides a very intuitive yet accurate way to describe cell signalling dynamics in a wide range of settings. The convenience of the discrete stochastic methodology presented in this paper is that, once the delay distributions are obtained, one can compute as many stochastic trajectories as necessary, while keeping computational times several orders of magnitude shorter than any spatially resolved method. Furthermore, delay models might succeed when mimicking directed transport mechanisms (by using an appropriate delay distribution) while particle tracking tools that do not support directed transport will fail.

We anticipate the use of our methodology will greatly aid the understanding of signalling pathways, incorporating non-negligible spatial effects in relatively fast simulations. For instance, new insights may be gained by revisiting well-known problems, such as the genetic toggle switch [28], by considering the effects of wide ranges molecular diffusion in gene expression, in relevant simulation time spans. Other direct applications may lie in the assessment of information transmission efficiency in signalling pathways limited by diffusion, such as the MAPK cascade.

## Conclusions

Biological systems are in many cases characterized by complex spatial structure, low diffusion rates, and low numbers of molecules, hence requiring spatially resolved simulations. However, these detailed spatially-resolved simulations can often only yield short simulation time spans that may not be of any interest to the experimentalists.

Here, we have presented effective ways of introducing spatial aspects into temporal models for a wide range of signaling scenarios and settings, yielding more accurate chemical kinetics in meaningful simulation times that are of actual biological interest. In such cases, we have shown that our discrete stochastic method achieves an accuracy that would never be attained using a solely temporal method, albeit at similarly low computational costs. Our research suggests that spatial heterogeneities can be well captured and modeled by means of time delayed processes with specific delay distributions, stemming from molecular diffusion profiles and the geometry of the cell and/or compartment analyzed. In some cases, this may provide new insights into complicated cellular processes and in a significantly shorter time frame than highly resolved spatial models. More research is needed in order to guarantee accuracy whenever two or more delayed reactions compete for a common reactant. Nevertheless, we hypothesize the consideration of fine-grained or theoretical time-varying delay distributions will greatly enhance accuracy whenever delays vary significantly, due to time or spatial restrictions (work in progress).

It is yet to be shown the cases and the extent to which our methodology could be incorporated into a coarse grained delayed simulator [29], achieving even shorter computational times.

## Methods

### ChemCell - a stochastic particle simulator

ChemCell is an off-lattice stochastic particle simulator developed at Sandia National Laboratories [1], where a cell can be represented as a collection of compartments with semi-permeable internal and external boundaries. Irrespective of molecular weight or chemical state, this software treats organic molecules as particles that diffuse via Brownian motion and are allowed to react with near-by particles in a probabilistic sense and with accordance to user-specified chemical reactions. Hence, the simulator time stepping procedure is divided into three stages: particle motion, neighbour finding and reactions.

#### Stage 1

Particles can be constrained to move within a compartment (3D) or a membrane (2D motion), or are allowed to translocate within compartments while considering user-specified membrane permeability. Permeability is defined as a cross-relation between each species and each membrane, ranging between the values 0 (impermeable) and 1 (fully permeable). The movement of a diffusing particle is considered to be the product of two/three 1D Gaussians, depending on whether the particle is constrained to diffuse within a membrane or compartment(s) and is independent of other particle's motions. The new coordinates of each particle are updated at each time step, upon which particles are tested to determine whether they are lying inside a new compartment. If so, a random number is generated to determine whether the particle will translocate to the new compartment, upon comparison with the user-specified permeability.

#### Stage 2

All molecule pairs closer to a pre-defined cut-off distance *R *will be considered as potential reaction partners, which will react with a probability *P *relative to the expected number of reactions happening during the time step (for details see [1]). So, the second stage of the time stepping procedure is achieved by binning the particles, where each bin's size depends on the cut-off value *R*. Consequently, two particles will be able to interact if and only if they lie in the same adjacent bins.

#### Stage 3

The last stage involves looping over the particles with reaction partners, for which a reaction will happen according to the above mentioned probability.

### Stochastic simulation algorithms for chemical reactions with delays

In the SSA the time between two reactions is regarded as the waiting time until the next reaction occurs, while reactions happen instantaneously. Unlike non-delayed reactions, delayed reactions trigger a state change at a future time point determined by the associated delay. In the implementation by Barrio et al. [12] the DSSA proceeds as the SSA as long as there are no delayed reactions scheduled within the next time step. Otherwise, it ignores the selected waiting time and rather continues from the scheduled update time point after updating the state according to the corresponding delayed reaction.

The algorithm separates waiting time and delay as this is a more natural representation of chemical kinetics. In the period between selection and update of a delayed reaction that consumes reactants other reactions can occur that consume the same reactants. By updating the delayed reaction this can lead to negative molecular numbers for the reactants. Therefore, reactants and products of delayed consuming reactions must be updated separately, namely when the delayed reaction is selected and when it is completed, respectively. In case a delayed reaction is non-consuming this aspect can be ignored. A more detailed description of the DSSA can be found in Barrio et al. [12].

## Authors' contributions

All authors participated in designing the study. TML performed ChemCell simulations, AL performed dDSSA simulations. TML and AL did the analysis. All authors drafted, read, and approved the final manuscript.
